# Improving PM_2.5_ prediction in New Delhi using a hybrid extreme learning machine coupled with snake optimization algorithm

**DOI:** 10.1038/s41598-023-47492-z

**Published:** 2023-11-29

**Authors:** Adil Masood, Mohammed Majeed Hameed, Aman Srivastava, Quoc Bao Pham, Kafeel Ahmad, Siti Fatin Mohd Razali, Souad Ahmad Baowidan

**Affiliations:** 1 Department of Civil Engineering, Jamia Millia Islamia University, New Delhi, India; 2 Department of Civil Engineering, Al-Maarif University College, Ramadi, Iraq; 3 Department of Civil Engineering, Indian Institute of Technology (IIT) Kharagpur, Kharagpur, 721302 West Bengal India; 4 Faculty of Natural Sciences, Institute of Earth Sciences, University of Silesia in Katowice, Będzińska Street 60, 41-200, Sosnowiec, Poland; 5 Department of Civil Engineering, Faculty of Engineering and Built Environment, Universiti Kebangsaan Malaysia, 43600 UKM Bangi, Selangor Malaysia; 6 Smart and Sustainable Township Research Centre (SUTRA), Universiti Kebangsaan Malaysia (UKM), 43600 UKM Bangi, Selangor Malaysia; 7 Green Engineering and Net Zero Solution (GREENZ), Universiti Kebangsaan Malaysia, 43600 UKM Bangi, Selangor Malaysia; 8 Information Technology Department Faculty of Computing and IT, King Abdulaziz University, Jeddah, Saudi Arabia; 9 Center of Excellence in Environmental Studies, King Abdulaziz University, Jeddah, Saudi Arabia

**Keywords:** Environmental sciences, Engineering

## Abstract

Fine particulate matter (PM_2.5_) is a significant air pollutant that drives the most chronic health problems and premature mortality in big metropolitans such as Delhi. In such a context, accurate prediction of PM_2.5_ concentration is critical for raising public awareness, allowing sensitive populations to plan ahead, and providing governments with information for public health alerts. This study applies a novel hybridization of extreme learning machine (ELM) with a snake optimization algorithm called the ELM-SO model to forecast PM_2.5_ concentrations. The model has been developed on air quality inputs and meteorological parameters. Furthermore, the ELM-SO hybrid model is compared with individual machine learning models, such as Support Vector Regression (SVR), Random Forest (RF), Extreme Learning Machines (ELM), Gradient Boosting Regressor (GBR), XGBoost, and a deep learning model known as Long Short-Term Memory networks (LSTM), in forecasting PM_2.5_ concentrations. The study results suggested that ELM-SO exhibited the highest level of predictive performance among the five models, with a testing value of squared correlation coefficient (*R*^2^) of 0.928, and root mean square error of 30.325 µg/m^3^. The study's findings suggest that the ELM-SO technique is a valuable tool for accurately forecasting PM_2.5_ concentrations and could help advance the field of air quality forecasting. By developing state-of-the-art air pollution prediction models that incorporate ELM-SO, it may be possible to understand better and anticipate the effects of air pollution on human health and the environment.

## Introduction

Air pollution is a significant problem that has far-reaching consequences on public health, the environment, and the economy. Fine particulate matter (PM_2.5_) is a critical air pollutant linked to chronic health problems and premature mortality in big metropolitans such as Delhi^1^. Accurate forecasting of fine particulate matter concentrations plays a crucial role in raising public awareness, enabling vulnerable groups to avoid exposure during periods of increased PM_2.5_ levels, and providing valuable information for public health alerts^2–5^. PM_2.5_ is a term generally associated with particulate pollutants having a diameter of 2.5 µm or less. These tiny particles may settle into the deepest parts of the human respiratory tract and cause severe health issues^6^. Industrial emissions, vehicle exhaust, and burning fossil fuels are primary sources of PM_2.5_, contributing to air pollution and potential health hazards^7^. Being the fifth most important risk factor for mortality in Delhi, particulate matter pollution is suspected of causing 10,000 premature deaths annually. This problem of PM_2.5-_based pollution poses a significant health risk to vulnerable populations, including children, the elderly, and individuals with preexisting medical conditions^8–11^.

In recent years, developing accurate and reliable models for predicting PM_2.5_ concentration has become an essential area of research that can help mitigate health risks associated with air pollution and improve public health^12^. Moreover, in literature, various modeling approaches, including chemical transport, statistical, machine learning, and deep learning models^13–15^ have been applied to predict PM_2.5_ concentration. Chemical transport models simulate the physical and chemical processes governing the transport and transformation of atmospheric pollutants^16^. The concentration of PM_2.5_ may be predicted using these models utilizing information on emissions, weather, and other environmental conditions. However, these models are computationally intensive and require high expertise to develop and run. Comparatively, the statistical models are simpler and more computationally efficient^17^. These models use statistical techniques to relate PM_2.5_ concentration to environmental variables such as temperature, humidity, and wind speed. However, these models are limited by their assumptions about the relationship between variables and may need to perform better under changing environmental conditions. In contrast to the abovementioned techniques, machine learning, and deep learning models use algorithms to identify patterns in large datasets. These models can be trained on historical data to predict PM_2.5_ concentration based on environmental variables^18^. Machine learning models have the advantage of being able to identify complex, nonlinear relationships between variables, and they can adapt to changing environmental conditions. However, a substantial amount of high-quality training data is needed to create and improve these models.

Machine Learning (ML) and Deep Learning (DL) models, such as Random Forest (RF), Long Short-Term Memory networks (LSTM), Support Vector Regression (SVR), Extreme Learning Machines (ELM), Extreme Gradient Boosting (XGBoost), Gradient Boosting Regression (GBR), etc., have been widely used to forecast PM_2.5_ concentrations. These models use historical data on air quality, meteorology, and traffic conditions to predict future PM_2.5_ concentrations. In a research done in Delhi, India, the RF model was applied to multi-stage modeling exercises using satellite data, land use factors, reanalysis-based meteorological variables, and population density to estimate PM_2.5_ concentrations^14^. The model outcomes indicated that the RF Model exhibited high prediction accuracy with yearly average concentrations ranging from 87 to 138 g/m^3^. Other researchers have reported comparable findings in applying ML and DL models to forecast PM concentrations for countries China, India, and the US^19^. In one of the studies, a DL model was developed for Delhi to forecast PM_2.5_ concentrations based on weather information and satellite images. The study found that the LSTM model outperformed other machine learning models statistically^12^. Moreover, a SVR model was applied for a study based on the city of Nottingham, UK to forecast PM_2.5_ concentrations using weather information and satellite data. The study found that the Multiple Linear Regression (MLR) and SVR models had good predictive performance compared to DL models^15^. In a similar study, an ELM model was utilized to forecast PM_2.5_ concentrations in London. It was concluded that the ELM outperformed the other conventional models, such as Artificial Neural Networks (ANN), regarding forecasting accuracy and performance^18^. Altogether, it can be proclaimed that the machine learning and deep learning models have shown promise in accurately forecasting PM_2.5_ concentrations.

Hybrid machine-learning algorithms have been extensively developed in the last decade for predicting PM_2.5_ concentrations^20–22^. These models have several advantages over standalone machine learning models for forecasting PM_2.5_ concentrations. These models have proven to enhance various critical aspects of performance, including accuracy, robustness, generalizability, and interpretability^23^. However, the process of choosing these models is inherently influenced by the unique requirements and constraints specific to each application. Compared to standalone models, hybrid machine learning models have demonstrated high flexibility, increased interpretability, and improved performance in terms of forecasting PM_2.5_ concentrations^24,25^. This is because they combine the strengths of multiple algorithms and can capture complex relationships between variables that a single algorithm may miss^26^. Since hybrid machine learning models can adapt to changes in the data by adjusting the weights of the algorithms, they are more robust to changes in input data than standalone models. For example, if there is a sudden change in meteorological conditions, a hybrid model can adjust the weights of the algorithms better to capture the impact of these changes on PM_2.5_ concentrations. Hybrid machine learning models are more generalizable than standalone models. This may be due to their ability to be trained on data from multiple sources, which allows them to capture the variability in PM_2.5_ concentrations across different regions. Hybrid machine learning models can be more interpretable than standalone models because they combine more interpretable algorithms, such as regression models, with less interpretable algorithms, such as neural networks.

Some researchers introduced the VAR-XGBoost model, combining Vector Autoregression (VAR), Kriging, and XGBoost for precise, continuous O_3_ concentration prediction^27^. The model was evaluated with ten-fold cross-validation and outperformed other models, including XGBoost, CatBoost, ExtraTrees, AdaBoost, RF, Decision Tree, Light Gradient Boosting Machine, etc. The study highlighted ozone’s strong correlation with PM_2.5_ and weak correlation with SO_2_, using China as a case study. Besides, other studies found that the meta-algorithms significantly improved the performance of the forecasting models hence obtaining higher forecasting results^28^. Furthermore, an innovative model combining Wavelet Transform (WT), Stacked Autoencoder (SAE), and Long Short-Term Memory (LSTM) has been introduced by some researchers for precise PM_2.5_ prediction^29^. The outcomes indicated that the predictive capability of SAE-LSTM surpasses that of other models, such as Back Propagation (BP), employed for comparison. This indicated that the hybrid model exhibited considerable promise in enhancing the accuracy of PM_2.5_ forecasts. In another study on developing a hybrid model, the scholars developed the CNN-LSTM model by merging the strengths of the “Convolutional Neural Network (CNN) and the Long Short-Term Memory (LSTM) neural network” to forecast PM_2.5_ concentration in Beijing over the next 24 h^30^. This approach optimized CNN for effective feature extraction related to air quality, while LSTM captured the extended historical context of the input time series data. For comparative analysis, four models, including univariate and multivariate LSTM and univariate and multivariate CNN-LSTM, were constructed for PM_2.5_ prediction. The outcomes revealed that the multivariate CNN-LSTM model excelled in terms of both low error rates and shorter training times, making it the most effective choice for PM_2.5_ concentration forecasting. Besides, in yet another study to enhance the accuracy of PM_2.5_ predictions, a hybrid model, CEEMDAN-COOT-VMD-JAYA-LSSVM, was introduced^31^. The research incorporated complete ensemble empirical mode decomposition with adaptive noise (CEEMDAN), variational mode decomposition optimized using the COOT algorithm (COOT-VMD), and least square support vector machine optimized by the JAYA algorithm (JAYA-LSSVM). In comparison to both single and hybrid models, the results of DM tests provided robust evidence of the superiority of the proposed model, achieving a 99% confidence level over the comparison models. Such investigation have allowed researchers to understand the relationships between variables contributing to PM_2.5_ concentrations^32–34^.

Researchers have traditionally focused on using either DL or hybrid models for predicting PM_2.5_ pollution levels, as both approaches demonstrate strong performance in simulating air pollution dynamics. However, previous studies examining air pollution prediction do not directly compare these two predictive models. Also, developing accurate and reliable models is crucial to mitigate health risks associated with PM_2.5_ concentrations in Delhi, where elevated levels of PM_2.5_ pollution cause significant annual fatalities and contribute to a complex interplay of factors that influence public health outcomes. This research proposes a novel hybrid machine-learning model called ELM-SO (Snake Optimization—Extreme Learning Machine) for forecasting PM_2.5_ concentrations. The ELM-SO model combines two powerful machine learning techniques, ELM, and the Snake Optimization algorithm, to enhance the accuracy of PM_2.5_ predictions. ELM is chosen due to its superiority over traditional Artificial Neural Networks (ANNs) as it trains faster, avoids local minima, and exhibits exceptional performance^35^. Furthermore, the Snake Optimization algorithm is employed to train the ELM, as it has proven effective in solving various engineering problems and outperforms other established and newly developed algorithms (e.g., Moth-flame Optimization, Harris Hawks Optimizer, Whale Optimization Algorithm, and others)^36^. The proposed model incorporates air quality inputs and meteorological parameters to capture the complex relationships between PM_2.5_ concentrations and environmental factors. To assess the prediction performance of the ELM-SO model, the study aims to validate its performance by comparing it against the standalone machine learning models such as Support Vector Regression (SVR), Random Forest (RF), Extreme Learning Machines (ELM), Extreme Gradient Boosting (XGBoost), and Gradient Boosting Regression (GBR).

This study makes a significant and novel contribution to environmental engineering and climate change research. It diverges from previous work that mainly focused on deep learning or hybrid models for PM_2.5_ pollution prediction by directly comparing these two approaches. Findings from the present investigation are immensely useful due to their practical implications. First, the findings from this study will contribute to the focused understanding of air quality dynamics, allowing for better comprehension of the complex relationships between environmental factors and PM_2.5_ pollution. This knowledge is essential for environmental engineers, policymakers, and public health officials to design effective strategies for mitigating air pollution and its health effects. Secondly, the proposed ELM-SO model, which enhances the accuracy of PM_2.5_ predictions, can be applied in real-time air quality forecasting systems. This means that residents, especially those in highly polluted areas like Delhi, can receive timely and accurate information about air quality. This information enables individuals to take protective measures, such as wearing masks or staying indoors during high pollution episodes, thereby safeguarding their health. Furthermore, decision-makers can use these findings to formulate evidence-based policies and regulations for reducing air pollution. By understanding the factors that influence PM_2.5_ concentrations, cities and governments can implement targeted measures to limit pollution sources, such as industrial emissions or vehicle traffic. This, in turn, will prove beneficial to both individuals and communities in their endeavors to alleviate the detrimental effects of compromised air quality.

## Material and methods

### Study area and available data

**Figure 1:**
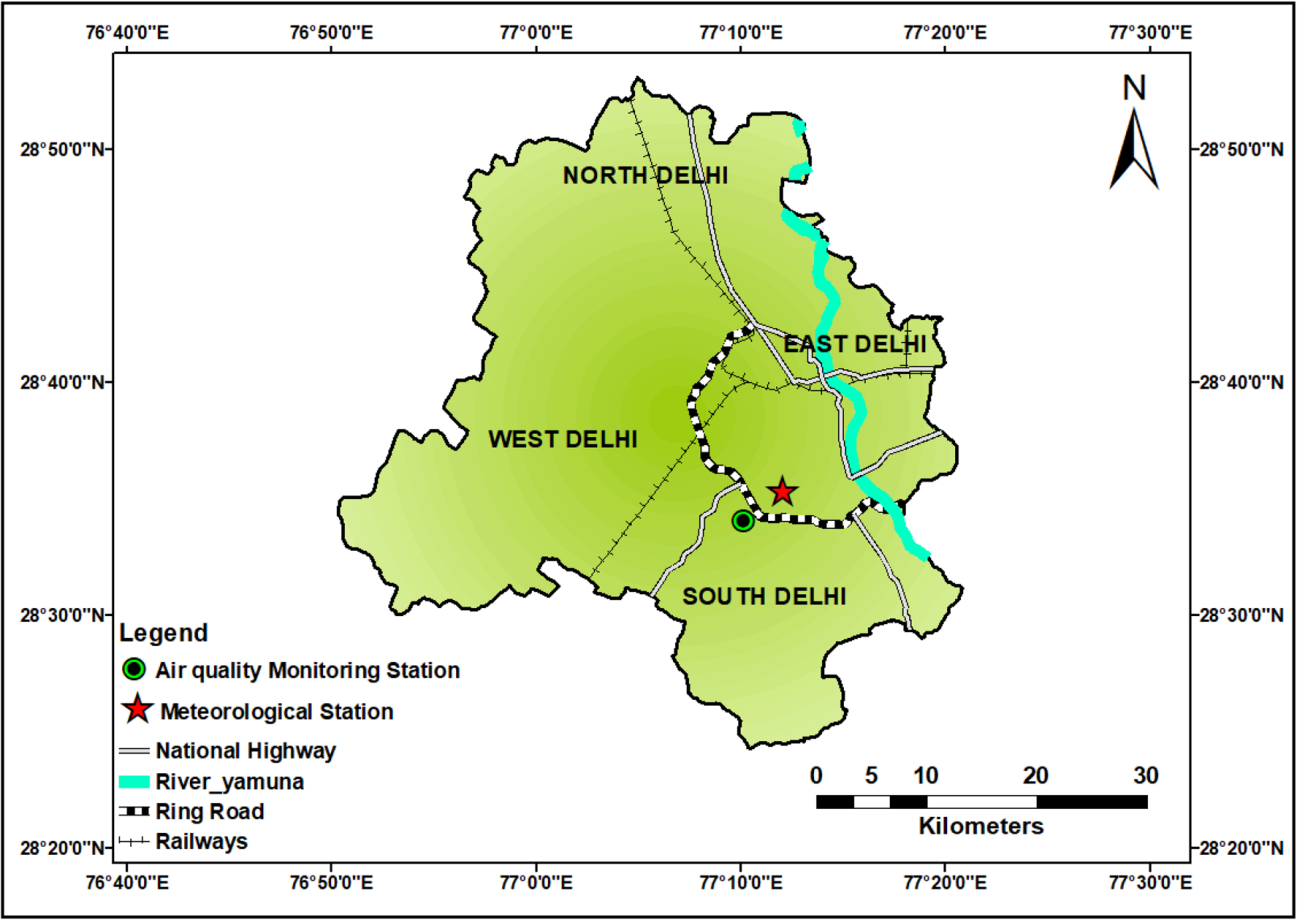
Air quality monitoring and meteorological stations.

The metropolitan city of Delhi, located in northeastern India, is one of the most rapidly expanding urban regions globally. The city spans an area of 1483 km^2^ and is situated within the geographic coordinates of north latitudes 28° 0.21′ to 28° 0.53′ and East longitudes 76° 0.20′ to 76° 0.37′, with an elevation of 216 m above mean sea level. Boasting a population of 16.75 million, the city experiences severe environmental stress in the form of poor air quality, putting its inhabitants' lives at risk^12^. The region has sub-tropical semi-arid (steppe) climatic conditions, characterized by an average annual precipitation of 611.8 mm and a mean annual temperature of 31.5 °C. Due to a combination of factors, including distinct geography, meteorological conditions, and rapid urban expansion, Delhi experiences some of the poorest air quality in the northern hemisphere. The city usually encounters an average of 15 pollution episodes each year, with over 200 days in a year exceeding the National Ambient Air Quality Standards (NAAQS) limits for PM_2.5_^37^. This high frequency of pollution episodes and PM_2.5_ exceedances underscores the severity of the air pollution problem in the city. A total of 731 observations of daily averaged air quality and meteorological data from 2015 to 2018 (covering a 4-year period) were obtained from the RK Puram (air quality monitoring station) and Safdarjung airport (meteorological station) (Fig. 1). Also, the meteorological parameters, such as wind speed, evaporation, air temperature, and rainfall, for the studied location are obtained from NASA's open-source Data Access Viewer (DAV) available at https://power.larc.nasa.gov/data-access-viewer/. The air quality data procured often contains missing and corrupted values resulting from factors such as natural disasters, sensor shutdowns, or system crashes. To address this issue, we utilized a multidirectional imputation technique presented by^38^ to estimate pollutant concentrations for the missing values. Following the imputation process, we performed *Z*-score normalization to eliminate outliers from the data. This normalization procedure transforms the data into a standardized distribution with a mean of 0 and a standard deviation of 1. The following equation has been used to compute the *Z*-score normalization:

1 $$Z=\frac{X-\overline{X}}{\sigma }$$

where *Z* is the normalized value, X is the input, $\overline{X }$ is the mean value of the dataset and $\sigma$ denotes the standard deviation. The characteristics of both the training and testing data sets have been presented in Table 1.

**Table 1:** Characteristics of the dataset used in the study (2015 to 2018).

Parameters	Units	Minimum		Maximum		Mean		Standard Deviation	
		Training	Testing	Training	Testing	Training	Testing	Training	Testing
PM_10_	µg/m^3^	36.05	39.38	874.64	650.12	265.47	259.78	139.67	140.19
NO	µg/m^3^	1.02	1.95	400.12	247.46	61.62	62.55	59.97	58.98
NO_2_	µg/m^3^	22.44	25.74	151.56	147.78	72.97	72.91	26.07	26.53
NH_3_	µg/m^3^	7.69	5.50	142.95	103.69	39.65	41.15	20.99	18.62
Benzene	µg/m^3^	0.16	0.29	30.31	27.03	6.68	6.96	5.73	5.99
Toluene	µg/m^3^	0.22	1.15	68.06	60.22	17.11	17.57	12.25	13.05
PM_2.5_	µg/m^3^	17.89	20.35	714.71	460.5	132.40	131.39	93.25	86.69
Temp.(max.)	°C	13.10	13.20	45.50	43.40	32.14	31.38	6.51	7.09
Temp.(min.)	°C	4.60	4.00	31.90	31.60	19.76	19.36	7.39	7.61
WS (avg.)	m/s	1.27	1.11	8.86	8.77	5.49	5.38	2.05	2.11
Rainfall	mm	0	0	68.60	93.80	1.21	2.83	5.30	10.56
Evaporation	mm	0.90	0	8.50	7.80	4.58	4.38	1.30	1.42
Humidity	g/m^3^	13.42	21.76	94.75	98	59.28	61.95	16.19	15.94

### ML techniques

#### SVR

**Figure 2:**
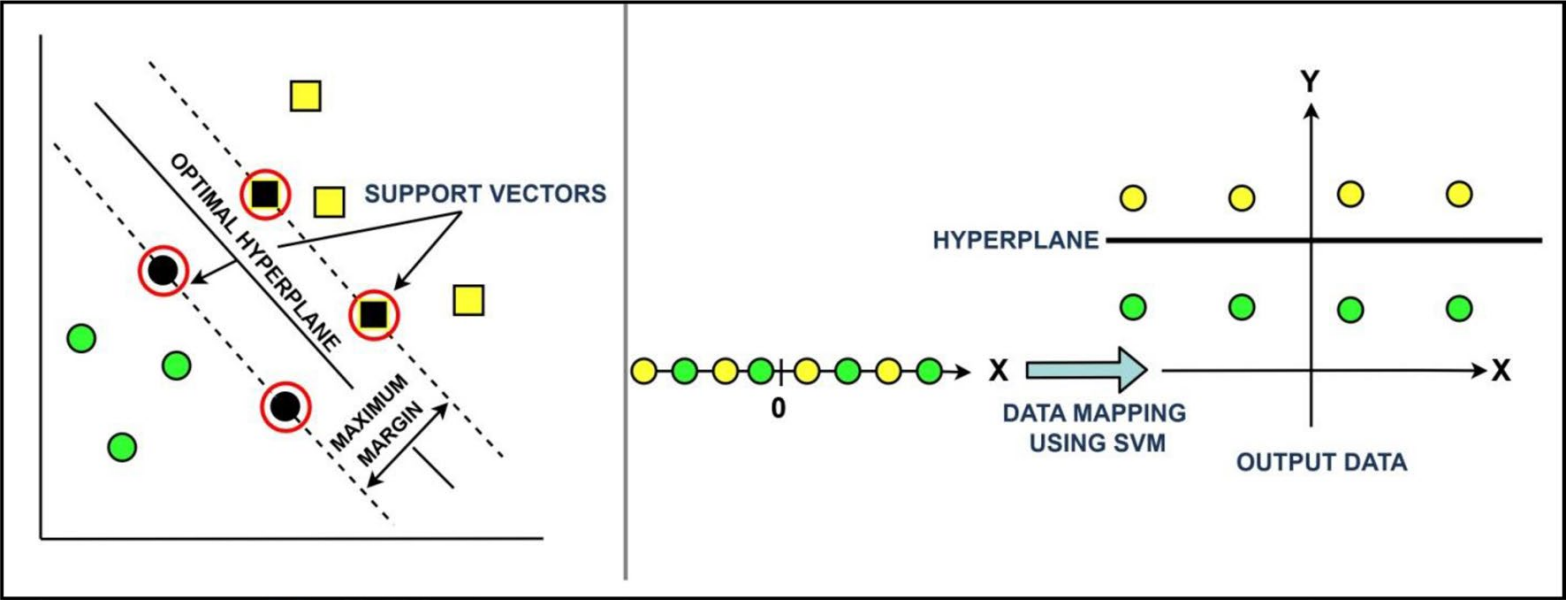
Support vector machine having an optimal hyper plane for classification.

Support Vector Regression (SVR) is a machine learning algorithm that can be used to predict PM_2.5_ levels based on a set of input parameters. Using a kernel function, the algorithm first transforms the input parameters into a higher-dimensional space^39,40^. In this new space, the algorithm then searches for a hyperplane that maximizes the margin between the predicted and the actual PM_2.5_ values in the training data (Fig. 2). To make predictions on new data, SVR maps the input parameters into the same higher-dimensional space as the training data and predicts the PM_2.5_ level based on its position relative to the hyperplane. The optimal hyperplane is determined by minimizing a cost function that penalizes errors in the predicted PM_2.5_ levels.

The input parameters used in the SVR model for PM_2.5_ prediction include temperature, wind speed, rainfall, evaporation, humidity, PM_10_, NO, NO_2_, NH_3_, benzene, and Toluene. These parameters are used to create a model that can accurately predict PM_2.5_ levels based on environmental conditions. The SVR can be formulated as follows:

2 $$Y \, = \, b \, + \, \sum \, a_{i} K\left( {x_{i} , \, x} \right)$$

where *Y* is the predicted value of PM_2.5_. *b* is the bias term. a_i_ is the coefficient of each input data point in the model. *x*_i_ is the input data point. *K(x*_*i*_, *x*) is the kernel function used to compute the similarity between the input data point *x*_i_ and the new data point x.

#### RF

Random Forest (RF) is a popular ensemble learning technique used for both regression and classification tasks. It functions by creating numerous decision trees during the training phase and determining the final prediction based on the mode of the classes (for classification) or the average prediction (for regression) from the individual trees^41,42^. In the RF model, each tree is constructed using a random subset of the training data and a random subset of the input features. The model then aggregates the predictions of all the individual trees to produce a final outcome^43^.

#### LSTM

The proposed model utilizes a Short-Term Long Memory (LSTM) neural network, which is a type of recurrent neural network (RNN), to predict PM_2.5_ concentrations based on input variables, including temperature, wind speed, rainfall, evaporation, humidity, PM_10_, NO, NO_2_, NH_3_, benzene, and Toluene. LSTM networks can handle temporal dependencies in the input data, making them suitable for prediction tasks such as air pollutant concentration forecasting^44^. The LSTM model's configuration is determined by its number of LSTM cells, hidden layers, and input/output dimensions. These LSTM cells are connected to both the input layer and each other through weighted connections, which are adjusted during the training phase. During this stage, the model is trained on a dataset using mean squared error (*MSE*) as the loss function, with the backpropagation algorithm updating the LSTM cell weights and optimizing the loss function. The model is then evaluated on a validation set using mean absolute error (*MAE*), mean squared error (*MSE*), and coefficient of determination (*R*^2^) as evaluation metrics. The trained LSTM model can then be used to predict the PM_2.5_ concentrations for new input data.

#### XGBoost

Extreme gradient boosting, or XGBoost, is a highly potent machine learning technique that relies on decision tree algorithms as its foundational unit of analysis. The technique generates a series of more powerful decision trees based on the forecasting inaccuracies of the preceding tree model. Unlike other ML models, XGBoost offers a higher level of complexity due to its numerous tunable parameters. While it shares some parameters with other tree-based models, XGBoost also necessitates additional hyperparameters. These hyperparameters are strategically designed to mitigate overfitting risk, minimize prediction variability, and thereby enhance overall prediction accuracy. XGBoost has the merit of effectively managing overfitting through the use of regularization which speeds up the model development process.

#### GBR

Gradient boosting regression tree is a machine learning approach that utilizes an ensemble of weak learners, usually decision trees, for forecasting. The algorithm iteratively enhances its predictions by adding new decision trees to the ensemble, with each tree trained to rectify the errors made by the preceding trees, resulting in increasingly accurate forecasts. Gradient boosting regression employs shallow decision trees as weak learners with minimal splits to prevent overfitting on training data. The technique can depict non-linear relationships, particularly those observed in various environmental systems, and deploys a variety of differentiable loss functions to learn throughout the iterations involving input features.

#### ELM

**Figure 3:**
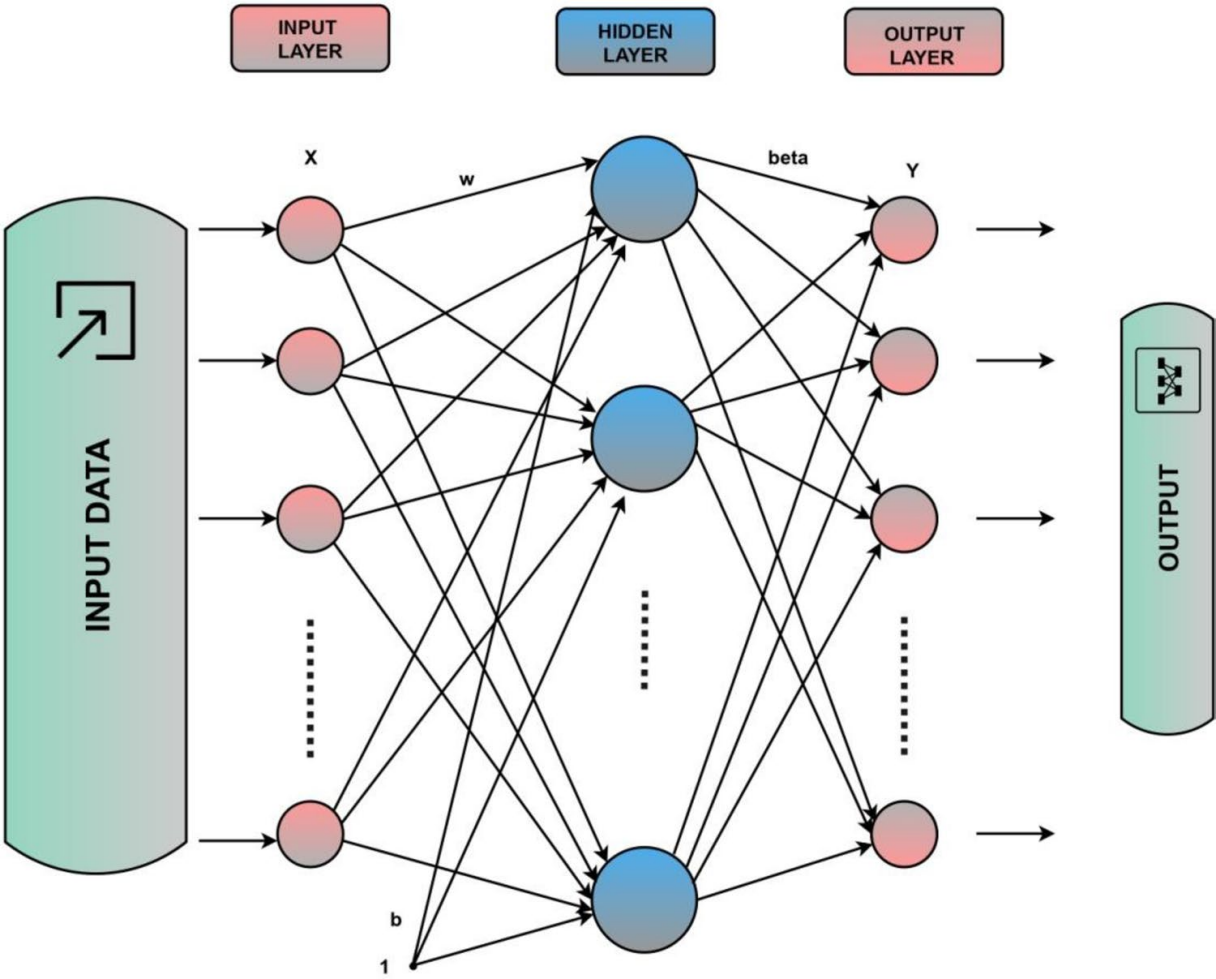
Typical configuration of an ELM.

Extreme Learning Machines (ELM) is a machine learning approach used for predicting PM_2.5_ values in this study. It is a feedforward neural network with a simple structure and fast processing operation^35^. During training, ELM uses many input neurons randomly selected from the available input features^45^. These input neurons are then connected to the hidden layer through randomly generated weights (Fig. 3). The output layer of the network is trained using the Moore–Penrose generalized inverse, which allows for fast and efficient training^46^. ELM is highly effective in predicting PM_2.5_ values, with superior performance compared to other machine learning methods. Additionally, ELM is highly scalable and capable of handling large amounts of data, making it an ideal choice for PM_2.5_ prediction tasks.

Given a set of training data {(xi, yi)} with xi ∈ Rn and yi ∈ R, ELM seeks to learn a mapping function f(x) = beta*(φ(x*w^T^)), where *w*^T^ and *φ*(*x*) are randomly generated weight and bias values and a nonlinear mapping function, respectively. Also, *beta* is output ELM weights. The mapping function *φ(x)* transforms the input features *x* into a high-dimensional feature space, where the nonlinear classification can be performed using a linear model with the weight vector *beta* (see Figure 3). In this work, sigmoid transfer function is used. The output weights are then calculated using the Moore–Penrose generalized inverse^47^:

3 $$beta= {\left({H}^{T}H\right)}^{-1}{H }^{T}y$$

Where *H* is the hidden layer output matrix, and y is the target output matrix, The trained ELM model can then be used to predict the PM_2.5_ values for new input data.

#### ELM-SO

The ELM-SO hybrid model combines the Extreme Learning Machines (ELM) algorithm with the Snake Optimization (SO) algorithm to improve the accuracy of PM_2.5_ prediction. The ELM-SO algorithm first uses the ELM algorithm to create a high-dimensional feature space and then applies the SO algorithm to select the optimal parameters of ELM for PM_2.5_ prediction.The SO algorithm is a meta-heuristic optimization algorithm that mimics the behavior of snakes searching for prey. The algorithm searches for the optimal subset of features by iteratively adjusting the snake's position in the search space. The following are the series of steps involved in the SO algorithm:

1. Initialization
  In the Snake Optimization Algorithm (SOA), the initialization phase involves generating a random population distributed uniformly within the search space^36^. This step allows the algorithm to commence the optimization process. The initial population is obtained using the following equation:
  4 $$P_{j} = P_{min} + R \times \left( {P_{max} - P_{min} } \right)$$
  where ${P}_{j}$ is the position of jth element, R represents a random number between 0 and 1, ${P}_{max}$ and ${P}_{min}$ establish the lower and upper limits of the problem, defining its respective boundaries.
2. Splitting of the population into two equal fractions: males and females
  In this step, an assumption is made where an equal distribution of males and females, each comprising 50% of the total population is carried out. The population is then split into two groups: a male group and a female group. To perform the division of the swarm, we employ the following equations:
  5 $${I}_{m}\approx I/2$$
  6 $${I}_{f}=I-{I}_{m}$$
  where *I* represents the total number of individuals, ${I}_{m}$ refers to the count of male individuals, and ${I}_{f}$ corresponds to the count of female individuals.
3. Assessing the two groups and establishing the optimal temperature and food quantity requirements
  Identify the top individual in each group and determine the best male, best female, and their respective positions in the food hierarchy
  The temperature (T) can be expressed by employing the following expression
    7 $$T=exp\left(\frac{{i}_{c}}{{i}_{T}}\right)$$
    In the above equation, ${i}_{c}$ represents the current iteration, while ${i}_{T}$ corresponds to the maximum number of iterations being considered.
  The Food quantity(F_q_) can be determined by using the following expression:
    8 $${F}_{q}={C}_{1}*\left(\frac{{i}_{c}-{i}_{T}}{{i}_{T}}\right)$$
  where, ${C}_{1}$ represents a constant whose value is considered as 0.5
4. Stage of Exploration (Lack of food)
  If ${F}_{q}$< Threshold, snakes randomly search for food and update their position accordingly using the following equation:.
  9 $$P_{j,m} \left( {i_{c} + 1} \right) = P_{rand,m} \left( {i_{c} } \right) \pm C_{2} \times M_{ab} \times \left( {P_{max} - P_{min} } \right) \times rand + P_{min}$$
  Here, ${P}_{j,m}$ denotes the position of the jth male, while ${P}_{rand,m}$ denotes the position of a randomly selected male, rand is a random number ranging from 0 to 1, while ${M}_{ab}$ denotes the male's ability to locate food. Similarly for the females, the position is updated with the following expression.
  10 $$P_{j,f} \left( {i_{c} + 1} \right) = P_{rand,f} \left( {i_{c} } \right) \pm C_{2} \times F_{ab} \times \left( {P_{max} - P_{min} } \right) \times rand + P_{min}$$
  Here, ${P}_{j,f}$ represents the position of the jth female, while ${P}_{rand,f}$ is the position of a randomly selected female, rand is a random number ranging from 0 to 1, while ${F}_{ab}$ denotes the female's ability to locate food.
  The ability of males or female individuals to look for food is given by the expression:
  11 $${M}_{ab}/{ F}_{ab}=\mathrm{ exp}(\frac{-{f}_{rand,m/rand f}}{{f}_{j,m/j,f}})$$
  where, ${f}_{rand,m/rand,f}$ indicates the fitness of ${P}_{rand,m}$ / ${P}_{rand,f}$,${f}_{j,m/j,f}$ represents the fitness of the jth individual in the male or female group, and ${C}_{2}$ is a constant with a fixed value of 0.05.
5. Stage of Exploitation (Food is present).
  If ${F}_{q}$ and T > Threshold, snakes only look for food and update their position accordingly using the following equation.
  12 $$P_{j,k} \left( {i_{c} + 1} \right) = P_{food} \pm C_{3} \times T \times rand \times \left( {P_{food} - P_{j,k} \left( {i_{c} } \right)} \right)$$

where,${P}_{j,k}$ indicates the individual location (male or female),${P}_{food}$ represents the best individuals position, and ${C}_{3}$ is a constant with a fixed value of 2.

T < Threshold (0.6) triggers the fighting or mating mode of the snake.

- Fight mode

13 $$P_{j,m} \left( {i_{c} + 1} \right) = P_{j,m} \left( {i_{c} } \right) + C_{3} \times MF_{ab} \times rand \times \left( {F_{q} \times P_{best,f} - P_{j,m} \left( {i_{c} } \right)} \right)$$

Here, ${P}_{j,m}$ represents the position of the jth male, while ${P}_{best,f}$ is the best individual female's position, rand is a random number ranging from 0 to 1, while ${MF}_{ab}$ denotes the male's ability to fight.

Similarly,

14 $$P_{j,f} \left( {i_{c} + 1} \right) = P_{j,f} \left( {i_{c} + 1} \right) + C_{3} \times FF_{ab} \times rand \times \left( {F_{q} \times P_{best,m} - P_{j,f} \left( {i_{c} + 1} \right)} \right)$$

Here, ${P}_{j,f}$ represents the position of the jth female, while ${P}_{best,m}$ is the best individual male's position, rand is a random number ranging from 0 to 1, while ${FF}_{ab}$ denotes the female's ability to fight.

- Mating mode

15 $$P_{j,m} \left( {i_{c} + 1} \right) = P_{j,m} \left( {i_{c} } \right) + C_{3} \times MM_{ab} \times rand \times \left( {F_{q} \times P_{j,f} - P_{j,m} \left( {i_{c} } \right)} \right)$$

16 $$P_{j,f} \left( {i_{c} + 1} \right) = P_{j,f} \left( {i_{c} } \right) + C_{3} \times FM_{ab} \times rand \times \left( {F_{q} \times P_{j,m} - P_{j,f} \left( {i_{c} } \right)} \right)$$

Here,${P}_{j,m}$ and ${P}_{j,f}$ represents the position of the jth male and female, while ${MM}_{ab}$ and ${FM}_{ab}$ denote the male and female's ability to mate.

When an egg hatches, the least performing male and female are selected and replaced

17 $$P_{worst,m} = P_{min} + rand \times \left( {P_{max} - P_{min} } \right)$$

18 $$P_{worst,f} = P_{min} + rand \times \left( {P_{max} - P_{min} } \right)$$

Here,${P}_{worst,m}$ and ${P}_{worst,f}$ denote the worst performing male and female of a population group.

The ELM-SO hybrid model offers several advantages over other prediction models, such as improved accuracy, reduced computational complexity, and increased noisy and missing data robustness. The combination of the ELM algorithm and the SO algorithm enables the model to efficiently identify the most informative features for PM_2.5_ prediction, resulting in more accurate and reliable predictions.

### Model development

This study developed a hybrid model called ELM-SO to predict PM_2.5_ levels at a critical station in India. This was achieved by combining the ELM and SO techniques with SO used to optimize the weights and biases of the ELM's hidden layer. In the proposed hybrid model, ELM-SO, the Snake Optimization (SO) algorithm is employed to optimize the parameters of the ELM with the objective of minimizing the root mean square error (RMSE) as the objective function. Initially, SO initializes the ELM parameters with random numbers. The algorithm then iteratively corrects and refines these parameters based on the mating behavior-inspired exploration and exploitation phases. Through dynamic adjustments of parameters, such as food quantity, temperature, and mating abilities, SO aims to reduce the objective function and improve the accuracy of the ELM model. This iterative optimization process enhances the robustness and adaptability of the ELM-SO hybrid model in predicting PM_2.5_ levels at the specified station in India.

**Figure 4:**
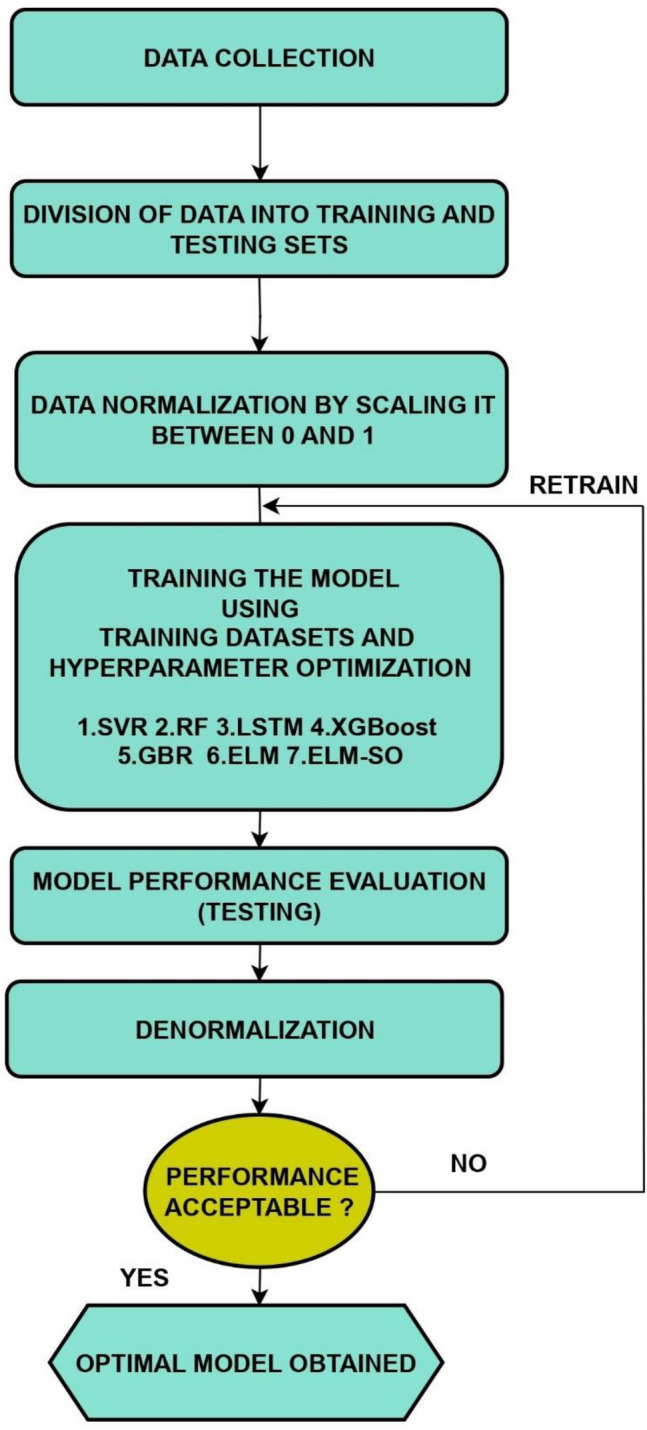
Modeling process of the ML models adopted in this study.

The hybrid model (ELM-SO) was validated against several other methods, including LSTM, RF, SVR, XGBoost, GBR and classic ELM. The data were divided into two stages: training, which involved using 75% of the total data to train the models and compute the best model parameters, and testing, which was used to check the model accuracy and select the best model. After dividing the data, the predictors and their corresponding values for PM_2.5_ are normalized between zero and one to improve the learning process of the ML models. In the case of the hybrid model, the SO is utilized to optimize the model's parameters by minimizing the error measured by the fitness function, which in this study is the root mean square error. The entire process of training the models is illustrated in Fig. 4, while the pre-set parameters for each model are provided in Table 2.

**Table 2:** Hyperparameters used for the developed models.

Technique	Hyper parameter
SVR	Box constraint = 1
	Epsilon = 7.268
	Kernel scale = 1
	Solver = SMO
	Iteration = 1000
LSTM	Gradient decay factor = 0.9
	Initial rate = 0.005
	Learning schedule = Piecewise
	Learning rate factor = 0.5
	Learning rate drop period = 102
	Gradient threshold = 0.1
	Max epochs = 250
	Minimum batch size = 128
RF	Fboot = 1
	Minimum leaf size = 7
	Number of trees = 12
ELM	Hidden nodes = 41
ELM-SO	Hidden nodes = 50
	Maximum iteration = 3000
	Number of search agent = 36
XGBoost	Maximum depth of a tree = 7
	Subsample ratio of the training instance = 0.1
	Subsample ratio = 0.75
	Minimum loss reduction = 0.08
	Lambda = 1
	Alpha = 0.1
GBR	Number of trees = 120
	Learning rate = 0.5

### Model performance metrics

The performance of the developed PM_2.5_ forecasting models in this study was evaluated with the help of five parameters: Root mean square error (*RMSE*), mean absolute error (*MAE*), Mean absolute percentage error (*MAPE*), Nash–Sutcliffe (*NSE*), Willmott Index (*d*), and *a20*-index. These metrics are mathematically defined as follows^48–51^:

19 $$RMSE=\sqrt{\frac{\sum_{i=1}^{N}{\left({I}_{p}-{I}_{o}\right)}^{2}}{N}}$$

20 $$MAE =\frac{1}{\mathrm{N}}\sum_{\mathrm{i}=1}^{\mathrm{N}}\left(\left|{\mathrm{I}}_{\mathrm{o}}-\overline{{\mathrm{I}}_{\mathrm{p}}}\right|\right)$$

21 $$MAPE= \frac{1}{\mathrm{N}}\sum_{\mathrm{i}=1}^{\mathrm{N}}\left(\left|\frac{{I}_{o}-{I}_{p}}{{I}_{o}}\right|\right)\times100$$

22 $$a20-index=\frac{{N}_{est}}{N}$$

23 $$NSE=1-\frac{\sum_{i=1}^{n}\left|{I}_{O}-{I}_{p}\right| }{\sum_{i=1}^{n}\left|{I}_{O}-\overline{{I }_{O}}\right|}$$

24 $$d =1- \frac{\sum_{\mathrm{i}=1}^{\mathrm{n}}({I}_{O}-{I}_{p}{)}^{2}}{\sum_{\mathrm{i}=1}^{\mathrm{n}}(\left|{I}_{p}- \overline{{I }_{O}}\right|{+ \left|{I}_{O}- \overline{{\mathrm{SSI} }_{\mathrm{obs}}}\right|)}^{2} }$$

where ${N}_{est}$ represents the count of estimates falling within the error range of ± 20% and *N* signifies the count of data points employed for the calculations, ${I}_{o}$ denote the actual data values and ${I}_{p}$ represent the predicted values.

## Uncertainty, reliability, and resilience analysis

This section evaluates the proposed methods through uncertainty analysis, reliability analysis, and resilience analysis of the RF, SVR, LSTM, GBR, XGBoost, ELM, and ELM-SO models. The uncertainty analysis aims to quantify and understand the level of uncertainty in the models' predictions, providing insights into the reliability and robustness of the proposed method. The U95 interval encompasses the range where the true value of the associated experiment outcome is estimated to occur about 95% of the time. The value of uncertainty (*U95*) can be determined using the following expression.

25 $$U95= \left(\frac{1.96}{N}\right)\sqrt{\sum_{j=1}^{N}{\left({{\left(Y\right)}_{j}}_{\left(observed\right)}-\stackrel{-}{{\left(Y\right)}_{\left(observed\right)}}\right)}^{2}+\sum_{j=1}^{N}{\left({{\left(Y\right)}_{j}}_{\left(observed\right)}-{{\left(Y\right)}_{j}}_{\left(predicted\right)}\right)}^{2}}$$

where* Y*_*observed*_ is the observed or actual value, *Y*_*predicted*_* is* the predicted value, and N is the number of data samples.

The reliability analysis assesses the consistency and accuracy of the models' outputs, understanding their performance under various conditions. The technique offers insights into the decision-making process, enabling informed decisions to be made on the necessary actions required to attain an acceptable level of performance for the model. The formula for estimating reliability is given by:

26 $$R= \left(\frac{100\%}{N}\right)\sum_{j=1}^{N}{O}_{j}$$

where *R* denotes the reliability and N is the number of data samples.

The value of Oj is computed using a two-stage process. First, the relative average error (RAE) is described as a vector whose jth component is

27 $${RAE}_{j}=\left|\frac{{{\left(Y\right)}_{j}}_{\left(observed\right)}-{{\left(Y\right)}_{j}}_{\left(predicted\right)}}{{{\left(Y\right)}_{j}}_{\left(observed\right)}}\right|$$

Next, if *RA*E_j_ ≤ ∆, then *Oj* = 1, else*, Oj* = 0, where ∆ denotes the threshold value. *Oj* is described as the number of times the value of *RAE *is less than or equal to *∆.*

Additionally, the resilience analysis investigates the models' ability to recover from inaccurate and unsatisfactory predictions. By analyzing the technique's performance under such conditions, resiliency analysis provides insights into its reliability and the extent to which it can adapt and rectify errors. The resiliency of model results is dependent on the reliability of model predictions. If the reliability of the model predictions meets a specific level, the resiliency will reach 100%. However, if the reliability falls below that level, the resiliency in the model results is calculated using the following equation:

28 $$\mathrm{Resiliency }=\frac{\sum_{\mathrm{j}=1}^{\mathrm{n}-1}{\mathrm{T}}_{\mathrm{i}}}{\mathrm{n}-\sum_{\mathrm{j}=1}^{\mathrm{n}}{\mathrm{O}}_{\mathrm{j}}}$$

where *T*_*i*_ represents the total cases in which the simulation has the probability of transitioning from an inaccurate prediction to an accurate forecast, indicating the model's ability to recover from initial errors.

## Results and discussions

### Model prediction results and comparison

Table 3 demonstrates the performances of deep learning, hybrid, and other ML models for forecasting PM_2.5_ concentrations. Among these, the hybrid model i.e., ELM-SO depicts the best forecasting capabilities, as evidenced by its lowest *MAE, RMSE, MAPE*, and highest *a20, NSE*, and *WI* values (*MAE *= 20.652 µg/m^3^, *RMSE* = 30.325 µg/m^3^, *MAPE* = 18.732%, *a20* = 0.688, *NSE* = 0.706 and *WI* = 0.972). The reason for this may be attributed to the ELM-SO model's ability to integrate the strengths of both SO and ELM. By doing so, the model can effectively tackle the issue of overfitting and prevent itself from being stuck in local optima. The XGBoost model, despite providing notable results, is the least performing model among the five models due to its lowest *a20* and *NSE* values (0.518 and 0.595) and highest *MAE* and *MAPE* values (28.439 and 30.792%).

**Table 3:** Performance metrics for PM_2.5_ forecasting models during the testing stage.

Models	RMSE (µg/m^3^)	MAE (µg/m^3^)	MAPE %	WI	NSE	a20
ELM	35.932	24.088	23.132	0.959	0.657	0.644
RF	36.061	23.603	22.148	0.953	0.664	0.660
SVR	38.359	26.248	24.672	0.948	0.627	0.603
LSTM	36.376	25.657	24.484	0.957	0.635	0.614
ELM-SO	30.325	20.652	18.732	0.972	0.706	0.688
XGBoost	38.290	28.439	30.792	0.951	0.595	0.518
GBR	35.299	23.923	20.808	0.962	0.660	0.641

**Figure 5:**
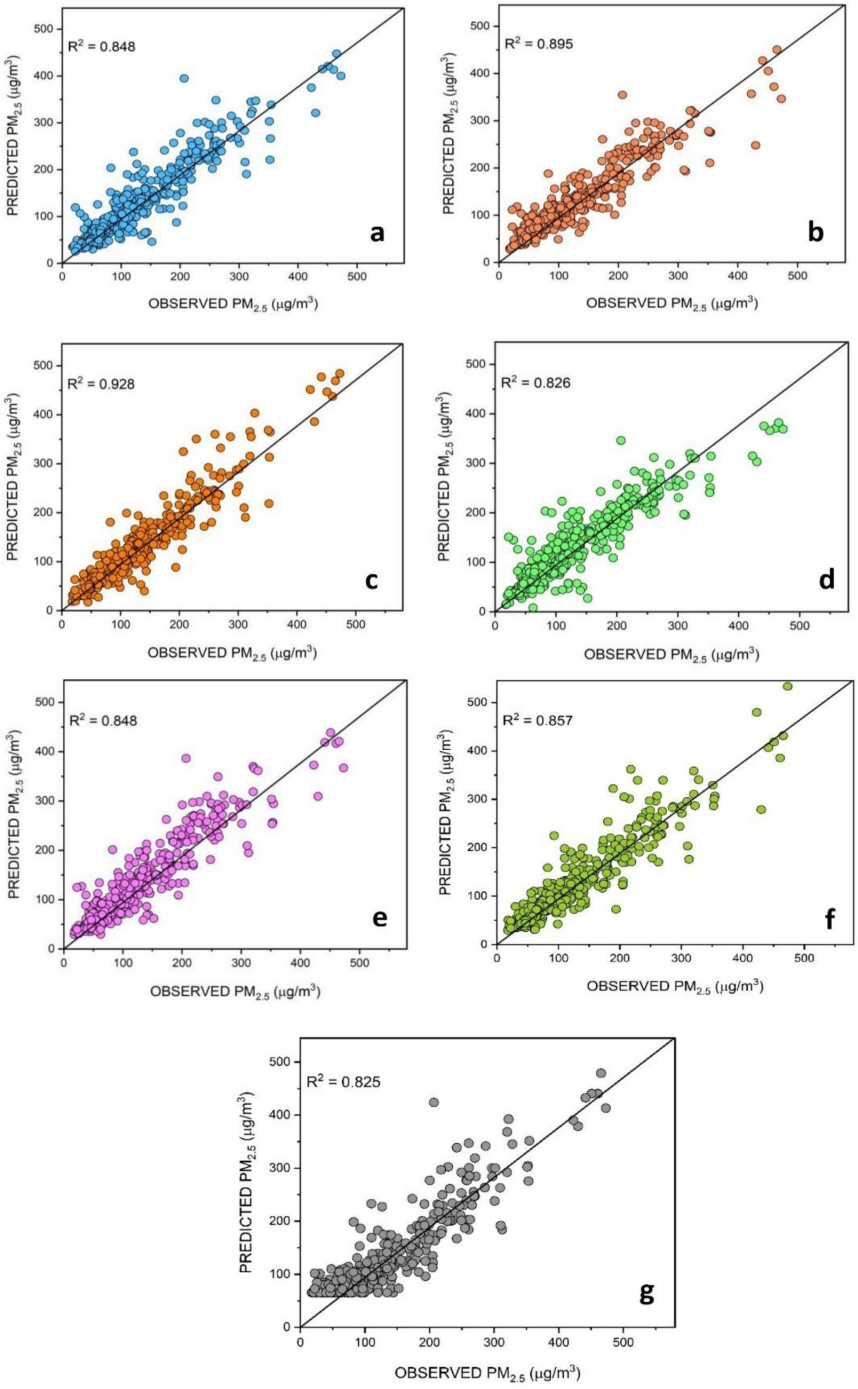
Scatter plots of the daily forecasted PM_2.5_versus. Observed PM_2.5_ concentrations for (**a**) ELM (**b**) RF (**c**) ELM-SO (**d**) SVR (**e**) LSTM, (**f**) GBR, and (**g**) XGBoost.

Figure 5a–g illustrates scatter plots featuring isoline regression lines and coefficient of determination (*R*^2^) to assess the correlation between observed and predicted PM_2.5_ for the testing phase. To ensure a high degree of model accuracy, the predicted and observed values need to be evenly distributed on both sides of the isoline regression line, indicating a Gaussian error distribution. This figure demonstrates that the hybrid model (ELM-SO) has a more favorable distribution of predicted values over the best-fit line and presents the highest *R*^2^ value (*R*^2^ = 0.928) compared to the other models. These observations derived from the scatter plot for the ELM-SO model align consistently with the trends presented in the statistical findings of our models (Table 3). This consistency reinforces the reliability and validity of the relationships and trends identified in our analysis, affirming the robustness of the model performance.

It is worth noting that, despite having satisfactory performances, the other individual models, i.e., ELM, RF, SVR, LSTM, GBR, and XGBoost fail to match the generalization capability of ELM-SO, and their accuracy and reliability get compromised for the testing dataset. The main factor contributing to this variability in the predictions is the non-linear nature of the predicted variable i.e., PM_2.5_ concentrations.

**Figure 6:**
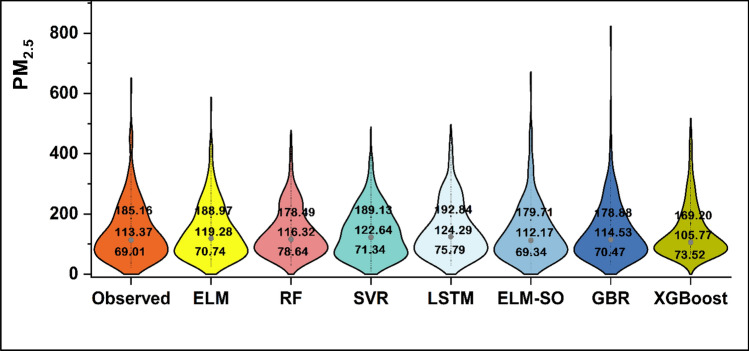
Violin plot for the observed and forecasted PM2.5 concentrations for the testing phase.

Figure 6 showcases the violin plots, which present a superior representation of data distribution, providing a clearer and more effective means of analysis. These plots evaluate the fidelity of the predicted PM_2.5_ concerning the observed values and compare the 25%, 50%, and 75% quantile values of both the experimental and predicted PM_2.5_, along with their corresponding interquartile ranges (IQR). From Fig. 6, it is clear that the LSTM (IQR = 117.05 µg/m^3^), SVR (IQR = 117.79 µg/m^3^), and ELM-SO (IQR = 110.37) based predictions correspond very closely to the observed data (IQR = 116.15 µg/m^3^). Furthermore, in the case of median value predictions, the ELM-SO and GBR models showed satisfactory performances with values of 112.17 µg/m^3^ and 114.53 µg/m^3^ compared to the observed median value of 113.37 µg/m^3^.

### Results of uncertainty, reliability, and resilience analysis

**Figure 7:**
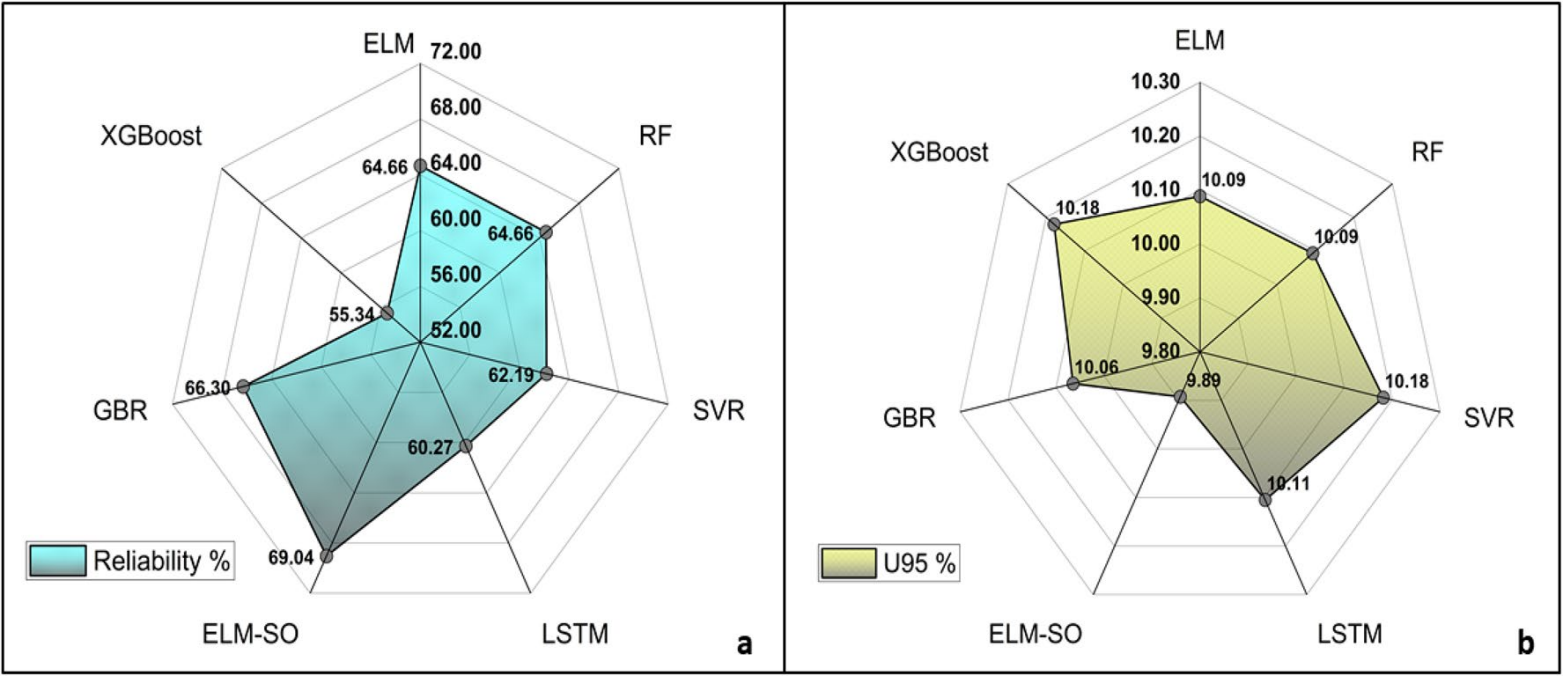
Radial plots depicting the (**a**) reliability and (**b**) uncertainty of all the developed models during the testing phase.

**Figure 8:**
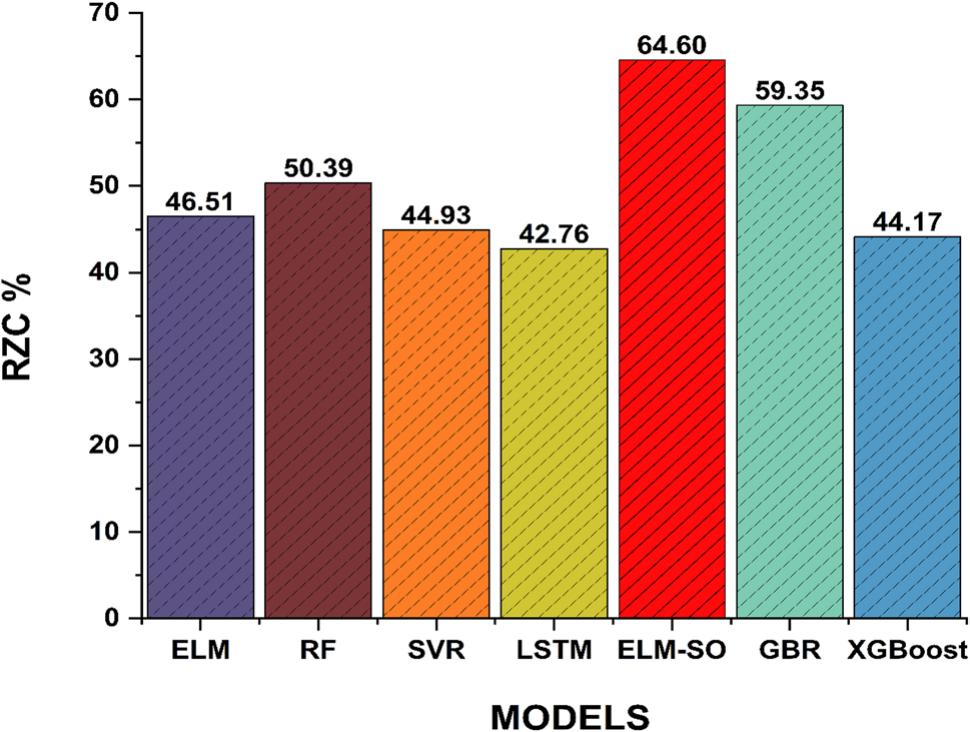
Bar chart depicting the resilience of all the developed models during the testing phase.

To assess the robustness of model results for the testing dataset, the uncertainty, reliability, and resilience indices are computed for the ELM, RF, SVR, LSTM, and ELM-SO models (Table 3 and Figs. 7 and 8). The results in the radar plot (Fig. 7b) indicate that ELM-SO exhibits the lowest uncertainty, with a *U95* value of 9.89, surpassing the performance of ELM (*U95* = 10.09), RF (*U95 *= 10.09), LSTM (*U95 *= 10.11), SVR (*U95* = 10.18), GBR (*U95* = 10.06) and XGBoost (*U95* = 10.18). The findings demonstrate ELM-SO's superior stability and consistency, indicating that ELM-SO is less sensitive in comparison to other ML models concerning the variations in the input data.

For the estimation of PM_2.5_ concentrations, the ELM-SO model showed more reliable forecasting performance with a reliability of 69.04% compared to other models. When compared to ELM (reliability = 64.66%), RF (reliability = 64.66%), SVR (reliability = 62.19%), LSTM (reliability = 60.27%), GBR (reliability = 66.30%) and XGBoost (reliability = 55.34%), the ELM-SO consistently outperformed them and showed better reliability for the testing dataset (Fig. 7a). The higher reliability of ELM-SO confirms the model’s accuracy and consistency, not just over time but also across different datasets, with an insignificant margin of error.

Lastly, the resilience analysis also validated the superior performance of the ELM-SO model. According to the results for resilience and other parameters summarized in Fig. 8, it is clearly observed that the PM_2.5_ predictions generated by the hybrid ELM-SO model are more resilient (64.60%) in comparison to the individual ELM (46.51%), RF (50.38%), SVR (44.92%), LSTM (42.759%), GBR (59.35%) and XGBoost (44.17%) models respectively. Overall, the findings indicate that the hybrid model (ELM-SO) is a highly effective and accurate technique in estimating PM_2.5_ concentrations across a broad range of environmental factors, demonstrating compelling evidence of its efficiency.

### Validation of ELM-SO with previous works

In our study, a novel hybrid ELM-SO model was developed to simulate PM_2.5_ concentrations. The results in terms of statistical metrics (*R*^2^, *RMSE, MAE, MAPE, a*_20_, *NSE*, and* WI*) revealed a substantial enhancement in performance compared to ELM, RF, LSTM, SVR, GBR, and XGBoost models. Some researchers developed a novel technique for PM_2.5_ forecasting by combining Genetic algorithm and Support vector machine^52^. The models were constructed using land use, meteorological, elevation, traffic, socioeconomic, vegetation, landscape pattern, AOD, and other data as inputs. The results showed that the proposed hybrid modeling approach achieved favorable accuracy (*R *^2^ = 0.840) compared to other individual models. Other scholars presented a novel method by combining spatial–temporal analysis with deep learning approach to estimate PM_2.5_ concentrations^53^. The model was constructed by incorporating air quality and meteorological data as inputs to enhance its accuracy and reliability. The results of the proposed hybrid model were evaluated with other modeling techniques such as XGBOOST, BPNN, and RF. The results indicated unsatisfactory performances of the comparable models, whereas the proposed model achieved the highest accuracy with an* R*^2^ value of 0.700.

Furthermore, a published scientific paper reported the development of a novel hybrid system based on Deep learning and Gradient boosting approaches for PM_2.5_ prediction^54^. The study incorporated the outdoor imagery dataset as inputs for the model generation. It was concluded that the proposed technique showed good accuracy (*R*^2^ = 0.85) enhancing the accessibility of PM_2.5_ concentration estimation for unobserved locations and time periods. Other investigations utilized a hybrid technique based on Random Forest and Kriging to investigate the PM_2.5_ prediction and its distribution^55^. The novel approach was developed using air quality, meteorological, land use, and AOD data. The accuracy of the proposed model was evaluated by comparing the PM_2.5_ with that generated by the individual RF model. The results demonstrated that the proposed hybrid model with an *R*^2^ = 0.881 outshines the individual RF model in terms of accuracy and performance. In another work, some researchers carried out a PM_2.5_ forecasting study using a novel prediction system based on a Convolutional block attention module, Convolutional neural networks, and bi-directional long short-term memory networks^56^. The results of the study revealed that the proposed model was able to accurately predict the PM_2.5_ concentrations with an *R*^2^ value of 0.8162. Table 4 summarizes the results of previous studies that developed different prediction models for simulating PM_2.5_ concentrations. The results show that the suggested model, ELM-SO, outperformed all other comparable models.

**Table 4:** A comparison of PM_2.5_ prediction models.

Reference	Model	R^2^ value
^52^	GA-SVM	0.840
^53^	SIDLM	0.700
^54^	CNN-GBM	0.850
^55^	RFSTK	0.881
^56^	CBAM-CNN-Bi LSTM	0.8162
^57^	XGBoost	0.761
^58^	MLR	0.766–0.875
^59^	ANN	0.896
Proposed model	ELM-SO	0.928

### Discussion

The research findings indicated that the combination of SO and ELM in a hybrid model performed significantly better than using a single ELM for predicting PM_2.5_ concentration. These results align with other scientific papers published in the literature, which have also shown that hybrid models provide improved prediction performance for simulating air pollution parameters^60–62^. Additionally, the SO algorithm enhances the prediction accuracy of the standard ELM by determining optimal weights and bias values. The evaluation statistical criteria and visualization assessments demonstrate that the hybrid model outperforms not only the classical ELM but also other well-known models frequently used in the air quality sector, such as RF, LSTM, XGboost, GBR, and SVR.

The SO algorithm effectively discovers global solutions by utilizing nature-inspired behavior, balancing exploration and exploitation, and demonstrating efficient convergence^36^. By mimicking the mating behavior of snakes, SO explores new solution spaces to find innovative and effective solutions. It efficiently converges to satisfactory solutions within a reasonable number of iterations, making it particularly valuable in time-sensitive applications. These characteristics make the suggested model advantageous in predicting PM_2.5_ more effectively compared six models developed in this study and other advanced models developed in previous research.

In the prediction of PM_2.5_, the integration of the SO algorithm with ELM offers several technical advantages. First, SO replaces random assignment to guide the optimization of weight and bias parameters in ELM, resulting in enhanced parameter optimization. Second, the algorithm mimics the behavior of snakes in its exploitation and exploration phases, enabling both local fine-tuning and global search capability. Besides, by dynamically adapting parameters such as food quantity, temperature, and mating abilities, the algorithm gains the ability to effectively navigate and explore complex optimization spaces. This adaptability empowers the algorithm to efficiently search for optimal solutions within intricate problem domains, improving its overall performance and robustness in tackling challenging optimization tasks. Overall, the technical aspects and advantages of the SO algorithm demonstrate its potential to effectively optimize ELM's parameters in solving real-world optimization challenges.

Accurate prediction of fine particulate matter (PM_2.5_) concentrations is crucial in metropolitan areas like Delhi due to its association with chronic health issues and premature mortality. The precise forecasting of PM_2.5_ levels serves multiple purposes, including raising public awareness, aiding vulnerable groups in avoiding exposure during periods of high PM_2.5_ levels, and providing valuable information for public health alerts. Our adopted models (ELM-SO) have demonstrated improved reliability, as evidenced by lower values of RMSE (30.325 µg/m^3^), MAE (20.652 µg/m^3^), MAPE (18.7%), and U95 (9.893). These results highlight the significance of more precise forecasting in mitigating the negative impacts of PM_2.5_ on the population in India. Additionally, the suggested system provides early warning information for environmental management and facilitates the creation of efficient strategies to decrease air pollutant emissions. Furthermore, it contributes to advancing research and application in diverse domains, including the study of health concerns associated with PM_2.5_ pollution.

## Conclusion

This study assessed the predictive capabilities of a novel hybrid model known as ELM-SO, which utilizes the SO, for forecasting PM_2.5_ concentrations. The performance of ELM-SO was compared to that of standalone machine learning models including ELM, SVR, RF, LSTM, GBR, and XGBoost. To comprehensively gauge its performance, we conducted a comparative analysis against various standalone machine learning models and other forecasting models developed in previous works. The results demonstrated that the ELM-SO models outperformed other models in predicting PM_2.5_ with high accuracy. Furthermore, the ELM-SO model, developed using a novel metaheuristic algorithm (SO) and ELM, performed significantly better than LSTM and other models, achieving a minimum forecasting error of *RMSE *= 30.325 µg/m^3^, which was 15.6% to 20.9% more accurate than other models. The findings highlighted the effectiveness of the SO algorithm in improving the performance of ELM in predicting PM_2.5_ concentrations, with an enhancement of 15.6% in forecasting accuracy compared to standard ELM.

Additionally, the uncertainty analysis indicated that the ELM-SO model had a superior capability to present the lowest level of uncertainty compared to the other models. This was evidenced by the fact that it produced the lowest U95 value of 9.89 and had a higher estimated reliability forecasting of approximately 70%. Overall, this study suggests that the ELM-SO hybrid model is a promising approach for accurately predicting PM_2.5_ concentrations, which is crucial for mitigating the harmful effects of air pollution on public health and the environment.

## Data Availability

Data are available upon requested from the corresponding author.
